# A neutrophil elastase-generated mature form of IL-33 is a potent regulator of endothelial cell activation and proliferative retinopathy

**DOI:** 10.1038/s12276-024-01279-y

**Published:** 2024-08-01

**Authors:** Shivantika Bisen, Shailendra Kumar Verma, Chandra Sekhar Mukhopadhyay, Nikhlesh K. Singh

**Affiliations:** 1 Integrative Biosciences Center, Wayne State University, Detroit, MI 48202 USA; 2 Department of Ophthalmology, Visual and Anatomical Sciences, School of Medicine, Wayne State University, Detroit, MI 48202 USA; 3 Center for Infectious Disease and Vaccine Research, La Jolla Institute for Immunology, San Diego, CA 92037 USA; 4 Department of Bioinformatics, School of Animal Biotechnology, Guru Angad Dev Veterinary and Animal Sciences University, Ludhiana, Punjab 141004 India

**Keywords:** Angiogenesis, Translational research

## Abstract

Human interleukin-33 (IL-33) is a 270 amino acid protein that belongs to the IL-1 cytokine family and plays an important role in various inflammatory disorders. Neutrophil proteases (Cathepsin G and Elastase) and mast cell proteases (tryptase and chymase) regulate the activity of IL-33 by processing full-length IL-33 into its mature form. There is little evidence on the role of these mature forms of IL-33 in retinal endothelial cell signaling and pathological retinal angiogenesis. Here, we cloned, expressed, and purified the various mature forms of human IL-33 and then evaluated the effects of IL-33_95-270_, IL-33_99-270_, IL-33_109-270_, and IL-33_112-270_ on angiogenesis in human retinal microvascular endothelial cells (HRMVECs). We observed that IL-33_95-270_, IL-33_99-270_, IL-33_109-270_, and IL-33_112-270_ significantly induced HRMVEC migration, tube formation and sprouting angiogenesis. However, only IL-33_99-270_ could induce HRMVEC proliferation. We used a murine model of oxygen-induced retinopathy (OIR) to assess the role of these mature forms of IL-33 in pathological retinal neovascularization. Our 3′-mRNA sequencing and signaling studies indicated that IL-33_99-270_ and IL-33_109-270_ were more potent at inducing endothelial cell activation and angiogenesis than the other mature forms. We found that genetic deletion of IL-33 significantly reduced OIR-induced retinal neovascularization in the mouse retina and that intraperitoneal administration of mature forms of IL-33, mainly IL-33_99–270_ and IL-33_109–270_, significantly restored ischemia-induced angiogenic sprouting and tuft formation in the hypoxic retinas of IL-33^–/–^ mice. Thus, our study results suggest that blockade or inhibition of IL-33 cleavage by neutrophil proteases could help mitigate pathological angiogenesis in proliferative retinopathies.

## Human IL-33 mature forms drive pathological retinal angiogenesis

Interleukin-33 plays a role in many diseases and biological processes. This study investigates how various IL-33 mature forms affects blood vessel creation in the eye, especially in eye diseases. The researchers used genetic and drug-related methods to study the effects of IL-33 on blood vessel formation in the eye, focusing on how it regulates cellular signaling. The research used both in vitro and in vivo methods to understand IL-33’s role in abnormal blood vessel growth, specifically in oxygen-induced eye disease, a model for diseases like premature retinopathy and some aspects of diabetic retinopathy. The research concludes that IL-33, especially its enzyme-processed forms, plays a key role in the development of proliferative retinopathies by promoting abnormal blood vessel growth in the eye. This new understanding of IL-33’s function could lead to new treatments for proliferative retinopathies. This summary was initially drafted using artificial intelligence, then revised and fact-checked by the author.

## Introduction

Interleukin-33 (IL-33) is a protein belonging to the IL-1 cytokine family and is produced by several cell types, including epithelial cells, endothelial cells, fibroblasts, and immune cells such as dendritic cells and macrophages^1^. Initial studies on IL-33 revealed that it is an alarmin released in the extracellular space in response to cellular damage or necrotic cell death and that its primary role is to warn the immune system of tissue injury following trauma or infection^2^. However, various studies have implicated IL-33 in different disease conditions, including asthma, allergic diseases, autoimmune disorders, and cardiovascular diseases^3,4^. IL-33 has been shown to regulate the function of immune cells, such as T helper 2 (Th2) cells, mast cells, and eosinophils, which are involved in allergic and inflammatory responses^1,3^.

IL-33 has been recognized for its involvement in various biological processes, including angiogenesis, which is the generation or growth of new blood vessels from the existing vasculature. Studies from our laboratory and others have shown that IL-33 regulates endothelial cell activation and angiogenesis^5–8^. In human endothelial cells, IL-33 stimulates von Willebrand factor (vWF) expression and angiogenesis in a concentration-dependent manner^9^. In diabetic mice, IL-33 enhances wound healing by enhancing extracellular matrix deposition and neovascularization^10^. Furthermore, elevated IL-33 expression promotes angiogenesis in hypoxic human pulmonary artery endothelial cells in a hypoxia-inducible factor (HIF)-1alpha-dependent manner^11^. On the other hand, studies have also suggested that IL-33 inhibits angiogenesis, particularly ocular angiogenesis^12^.

**Fig. 1:**
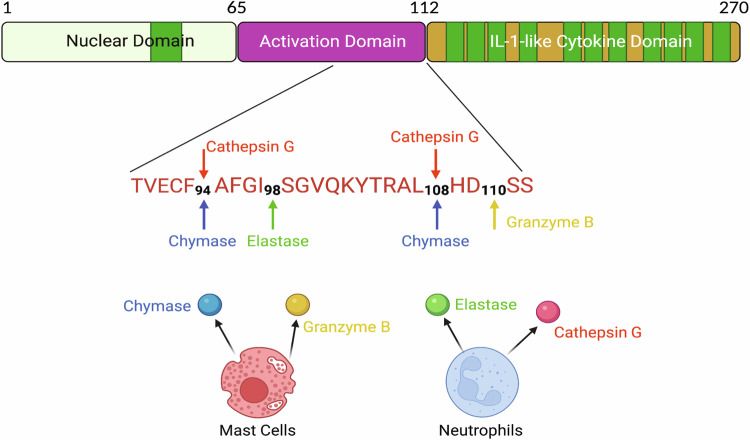
The primary structure of human IL-33. The human IL-33 protein consists of a nuclear localization domain (amino acids 1–65), an activation domain (amino acids 66–111), and an IL-1-like cytokine domain. The IL-33 protein sequences surrounding the cleavage sites for mast cell proteases (chymase and granzyme B) and neutrophil cell proteases (elastase and cathepsin G) are shown. The figure was created with BioRender.com.

Despite these significant improvements in understanding the roles of IL-33, little is known about the mechanisms that control IL-33 activity. The human IL-33 protein is localized in the nucleus of cells, where it binds to chromatin and the histone H2A-H2B via a short chromatin-binding motif (amino acids 40-58) (Fig. 1)^13–15^. Initially, caspase-1 cleavage of IL-33 into its mature form was believed to be essential for its biological activity^3^. However, it has been shown that IL-33 is not a physiological caspase-1 substrate; instead, it is cleaved by caspase-3 and caspase-7 at amino acids 175 and 178, respectively^2,16,17^. Furthermore, it has also been demonstrated that IL-33 processing by caspases leads to its inactivation as opposed to its activation^2,6,18^. Although caspase cleavage renders IL-33 inactive, numerous studies have demonstrated the existence of additional mechanisms that increase IL-33 activity^19,20^. Specific inflammatory cell proteases, such as neutrophil proteases (cathepsin G and elastase), as well as mast cell proteases (chymase and tryptase), have been shown to cleave IL-33 and yield mature 18–21 kDa fragments with a C-terminal IL-1-like cytokine domain^21^. The mature forms of IL-33 produced by neutrophil proteases (IL-33_95–270_, IL-33_99–270_, and IL-33_109–270_) and mast cell proteases (IL-33_95–270_ and IL-33_109–270_) have 30- to 60-fold greater biological activity than full-length IL-33^19,20^.

Several studies have highlighted the significance of inflammatory molecules in proliferative retinopathies^3,21,22^. Our previous findings demonstrated increased retinal expression of IL-33 in a mouse model of proliferative retinopathy^6^. Therefore, in the present study, we aimed to evaluate the angiogenic potential of mature forms of IL-33 generated by neutrophil and mast cell proteases on postischemic pathological angiogenesis. Here, we investigated the effects of IL-33_95–270_, IL-33_99–270_, IL-33_109–270_, and IL-33_112–270_ (mature forms of IL-33) on endothelial cell proliferation, migration, sprouting, tube formation and the angiogenic pathways they regulate in human retinal microvascular endothelial cells (HRMVECs). We provide evidence that the mature forms of IL-33 generated by neutrophil and mast cell proteases, particularly IL-33_99–270_ and IL-33_109–270,_ were more potent than the full-length form in regulating hypoxia/ischemia-induced angiogenic sprouting and tuft formation in the retina in a murine model of oxygen-induced retinopathy (OIR). Thus, our results demonstrate that proteolytic processing by inflammatory proteases induces IL-33 activity in proliferative retinopathies.

## Materials and methods

### Reagents

Anti-phospho serine/threonine (pSer/Thr, #9631, dilution 1:1000), anti-phospho extracellular signal-regulated kinase 1/2 (pERK1/2, #4370, dilution 1:1000), anti-ERK (#4695, dilution 1:1000), anti-phospho Jun-amino-terminal kinase 1/2 (pJNK1/2, #92515, dilution 1:1000), anti-JNK (#9252, dilution 1:1000), anti-phospho P38 mitogen activated protein kinase (pP38 MAPK, #4511, dilution 1:1000), anti-P38 MAPK (#9212, dilution 1:1000), anti-β-tubulin (#2128, dilution 1:1000), anti-delta-like protein 1 (DLL1, #2588, dilution 1:1000), anti-Jagged1 (#2620, dilution 1:1000), anti-Jagged2 (#2210, dilution 1:1000), anti-neurogenic locus notch homolog protein 1 (Notch1, #3608, dilution 1:1000), anti-Cleaved Notch1 (#4147, dilution 1:1000), anti-Notch2 (#5732, dilution 1:1000), anti-Notch3 (#5276, dilution 1:1000), anti-phospho nuclear factor kappa B (pNF-κB, #3033, dilution 1:1000), anti-NF-κB (#8242, dilution 1:1000), anti-phospho inhibitory kappa B (κB) kinase alpha/beta (pIKKα/β, #2697, dilution 1:1000), anti-IKKβ (#8943, dilution 1:1000), anti-phospho inhibitory kappa B alpha (pIκBα, #2859, dilution 1:1000), anti-IκBα (#4814, dilution 1:1000), anti-Fos-related antigen 2 (Fra2, #199967, dilution 1:1000), anti-FosB (#2251, dilution 1:1000), anti-JunD (#5000, dilution 1:1000), anti-a disintegrin and metalloproteinase domain-containing protein 10 (Adam10, #14194, dilution 1:1000), anti-intercellular adhesion molecule 1 (ICAM1, #4370, dilution 1:1000), anti-vascular cell adhesion molecule 1 (VCAM1, #4695, dilution 1:1000), and anti-E-Selectin (#92515, dilution 1:1000) antibodies were obtained from Cell Signaling Technology (Beverly, MA). Anti-Notch4 (ab184742, dilution 1:1000), anti-DLL4 (ab183532, dilution 1:1000), and anti-Ki-67 (ab15580, dilution 1:100) antibodies were purchased from Abcam Biotechnology Company (Cambridge, UK). Anti-Fra1 (sc-28310, dilution 1:500), anti-cFos (sc-166940, dilution 1:500), anti-cJun (sc-74543, dilution 1:500), and anti-JunB (sc-8051, dilution 1:500) antibodies were purchased from Sant Cruz Biotechnology (Dallas, Texas). Anti-ST2 (AF523-SP, dilution 1:500) and anti-IL-33 (AF3626, dilution 1:1000) antibodies and recombinant IL-33 (3625-IL-010/CF) protein were purchased from R&D Systems (Minneapolis, MN). Growth factor-reduced Matrigel (354230) was purchased from BD Biosciences (Bedford, MA). Endothelial cell growth medium 2 (EGM2) was purchased from Lonza (Basel, Switzerland). TRIzol reagent (15596026) was also purchased from Ambion (Carlsbad, CA). VECTASHIELD Antifade Mounting Medium with DAPI (4′,6-diamidino-2-phenylindole, #H-1500) and without DAPI (#H-1700) were purchased from Vector Laboratories (Burlington, Ontario, Canada). An anti-Adam17 antibody (703077, dilution 1:1000), ProLong Gold antifade reagent (P36984), Hoechst 33342, Alexa Fluor 488-conjugated goat anti-rat immunoglobulin G (A11006, dilution 1:250), Alexa Fluor 568-conjugated goat anti-rabbit immunoglobulin G (A11006, dilution 1:250), Cell Tracker Green (C7025), and isolectin B4-594 were purchased from Invitrogen (Carlsbad, CA). A BrdU flow Kit (51-2354AK) and an anti-CD31 antibody (550274, dilution 1:100) were purchased from BD Bioscience (San Jose, CA). Anti-matrix metalloproteinase-2 (MMP-2, #IM33, dilution 1:100) and anti-Tyr (#5777, dilution 1:1000) antibodies were obtained from MilliporeSigma (Rockville, MD).

### Cloning the IL-33 constructs into pET30a

Genomic DNA was isolated from human retinal endothelial cells using a commercially available kit (Qiagen, Germany). The IL-33_95–270_ construct was amplified by PCR using the forward 5′- ATTCATATGGCCTTTGGTATATCAGGGGT-3′ and reverse 5′-TAACTCGAGAGTTTCAGAGAGCTTAAACAAG-3′ primers. The IL-33_99–270_ construct was amplified by PCR using the forward 5′- ATTCATATGTCAGGGGTCCAGAAATATACTA-3′ and reverse 5′- TAACTCGAGAGTTTCAGAGAGCTTAAACAAG-3′ primers. The IL-33_109–270_ construct was amplified by PCR using the forward 5′- ATTCATATGCATGATTCAAGTATCACAGGAT-3′ and reverse 5′- TAACTCGAGAGTTTCAGAGAGCTTAAACAAG-3′ primers. The PCR amplicon was cloned and inserted into the pET30a vector using the restriction sites NdeI and XhoI (underlined nucleotide sequence). The pET30a plasmids carrying the in-frame IL-33 constructs were transformed into DH5α cells. The positive clones were screened in the presence of kanamycin (50 μg/ml) on Lysogeny Broth (LB)-agar plates.

### Expression of recombinant proteins

One of the positive clones corresponding to each recombinant construct (IL-33_95–270_, IL-33_99–270_, and IL-33_109–270_) was selected randomly and transformed into chemically competent BL-21 (DE-3) cells. The positive clones were screened on the LB-agar kanamycin plates. One of the positive clones corresponding to each recombinant construct was inoculated in 5 ml of LB broth containing kanamycin and grown overnight at 37 °C. One percent of the overnight culture was inoculated into 5 ml of kanamycin-containing LB broth and grown at 37 °C until the OD600 reached 0.6–0.8. At this time point, 1 ml of culture from each clone was centrifuged, and the pellet was stored at −20 °C as an uninduced pellet. The remaining 4 ml of each culture was induced with 1 mM isopropyl β-D-1-thiogalactopyranoside (IPTG) and grown further for 4 h. All the cultures from each clone were aliquoted in 1.5 ml tubes (1 ml culture) and pelleted as described earlier, and all the tubes were marked as induced and stored at −20 °C until subsequent use. The cultures were lysed in 1X sample buffer and subjected to sodium dodecyl sulfate‒polyacrylamide gel electrophoresis (SDS‒PAGE).

### Purification of recombinant proteins

The recombinant proteins were purified by affinity chromatography using Ni-NTA columns (Qiagen, Germany). Briefly, positive clones for each IL-33 construct were grown overnight in 5 ml of kanamycin-containing LB broth at 37 °C and in 500 ml of LB broth thereafter. The cultures were then induced with IPTG and grown for 4 h. All the cultures were pelleted and resuspended in 20 ml of lysis buffer (50 mM NaH_2_PO_4_, 250 mM NaCl; 10 mM imidazole; pH 8.0). The solutions were individually sonicated and centrifuged, and the supernatants were collected. Supernatants containing the individual recombinant proteins were loaded onto Ni-NTA columns and allowed to pass through the column, which was then washed with wash buffer (250 mM NaCl, 50 mM NaH_2_PO_4_, 30 mM imidazole; pH 8.0). The recombinant IL-33 mature proteins were eluted using 15 ml of elution buffer (250 mM NaCl, 50 mM NaH_2_PO_4_, 200 mM imidazole; pH 8.0). SDS‒PAGE was used to evaluate the collected fractions for each protein. The purified protein fractions were subjected to dialysis using dialysis buffer (50 mM NaCl, 50 mM NaH_2_PO_4_; pH 8.0). The purities of all three prepared recombinant IL-33 proteins were analyzed by SDS‒PAGE.

### Experimental animals

The Charles River Laboratories provided us with C57BL/6 mice (Wilmington, MA). The Jackson Laboratory provided the E2a-Cre (003724) and IL-33^flox/flox^ (030619) mice (Bar Harbor, ME). The mice were housed under a 12-h light/12-h dark cycle with unlimited access to food and water. For this study, male and female mouse pups from postnatal Day 12 (P12) to P17 were utilized. The animal experiments were approved by Wayne State University’s Institutional Animal Care and Use Committee (IACUC), Detroit, MI.

### IL-33 knockout mice

Crossbreeding IL-33^flox/flox^ mice^23^ with E2a-Cre mice resulted in the generation of IL-33 knockout mice. In mice, the E2a-Cre recombinase^24^ resulted in germline deletion of IL-33.

### Cell culture

The HRMVECs were obtained from Cell Systems (ACBRI 181, Kirkland, WA). The cells were grown in a microvascular endothelial cell growth medium supplemented with antibiotics [gentamycin (10 μg/mL) and amphotericin B (0.25 μg/mL)] and maintained at 37 °C in a 5% CO_2_ atmosphere. HRMVECs were synchronized in serum-free media for 24 hours prior to the experiments to achieve quiescence.

### Wound healing assay

A wound healing assay was used to measure cell migration, as described previously^25^. Briefly, cells were cultured in six-well culture dishes and incubated for 24 h at 37 °C in serum-free endothelial cell growth medium. A straight-edged wound with a cell-free zone was made in each well using a sterile plastic micropipette tip. HRMVECs were grown in 6-well culture dishes and incubated for 24 h at 37 °C. A straight-edged wound was created with a sterile micropipette tip. The cells were washed and treated for 24 h with or without IL-33 (20 ng/mL) in serum-free medium containing 5 mM hydroxyurea. Cell migration was imaged using a Thermo Fischer Scientific EVOS M5000 microscope (Waltham, MA). NIH ImageJ version 1.43 was used to analyze the data. Cell migration was calculated as the percentage of wound healing [total wound area (0 h) − area (after 24 h)/total wound area 100].

### Spheroid assay

A three-dimensional spheroid assay with the HRMVECs was carried out using the methods described by Roy et al.^26^. To prepare spheroids (~400 cells per spheroid), the HRMVECs were labeled with 10 μM BCECF-AM, and 80,000 cells were diluted in 4 mL of Dulbecco’s modified Eagle’s medium (DMEM) supplemented with 10% fetal bovine serum (FBS) and 1% penicillin/streptomycin containing 20% Methocel. Using an eight-channel pipette, we pipetted 25 μl of the cell suspension dropwise onto a 150 mm cell culture plate. The plate was incubated upside down in a humidified incubator set at 37 °C with continuous 5% CO_2_ for 24 h to form spheroids. The hanging drops containing the spheroids were washed with phosphate-buffered saline (PBS), after which the spheroid suspension was transferred to a 15 ml conical tube and centrifuged at 200 × *g* for 5 min. The supernatant was aspirated, and 2 ml of ice-cold Methocel containing 20% FBS was added.

Separately, a collagen stock solution (4 ml) was prepared by adding 1 mg/ml collagen to ice-cold DMEM, and the pH was adjusted by adding 5 μl of 5 N NaOH. The collagen solution was added to the ice-cold Methocel containing the spheroids, and 250 μl of the solution with spheroids was pipetted into each well of a 24-well cell culture plate. The plate was incubated in a humidified CO_2_ incubator (5%) at 37 °C for an hour to polymerize and solidify the drops. After that, 250 μl of DMEM supplemented with 20 ng/ml of the recombinant IL-33 proteins was added to each well of the 24-well plate containing the collagen bed containing the implanted spheroids. The plate was then incubated in a humidified incubator for one to two days at 37 °C with 5% CO_2_. The spheroid images were captured using a confocal microscope (Zeiss LSM 800) with Zen’s image analysis software.

### Tube formation assay

Growth factor-reduced Matrigel was used for the tube formation assay, as previously described^6,7,25^. Briefly, a 24-well plate was coated with Matrigel, and then, 1 × 10^5^ quiescent HRMVECs were seeded onto the Matrigel-coated plate. The cells were incubated for six hours at 37 °C with or without agonist, and tube formation was monitored with an EVOS M5000 microscope. NIH ImageJ version 1.43 was used to calculate the tube length (measured in micrometers).

### BrdU cell proliferation assay

The BrdU concentration was measured using the manufacturer’s standard protocol (BD Biosciences). Quiescent HRMVECs were exposed to IL-33 (20 ng/mL) for 24 h. After 24 h, BrdU (10 μl/mL, 1 mM) was added to the cells, which were then incubated for 4 h at 37 °C. Following fixation and permeabilization using BD Cytofix/Cytoperm buffer, the cells were thoroughly washed with 1× BD perm/wash buffer. The cells were then again permeabilized, washed and incubated with DNase solution for 1 h at 37 °C. After washing, the cells were incubated for 20 min at room temperature (RT) with fluorescein isothiocyanate (FITC)-labeled anti-BrdU antibody. After the cells were washed, 20 μl of 7-aminoactinomycin D (7-AAD) solution was added, and the cells were resuspended in 1 ml of staining buffer. Next, the samples were examined using BD LSRII flow cytometer, BD Biosciences.

### Western blotting

An equivalent amount of protein from cells or tissue extracts was resolved on SDS‒PAGE gels, after which the proteins were transferred to a nitrocellulose membrane. The nitrocellulose membranes with transferred protein were blocked (5% (w/v) nonfat dry milk or bovine serum albumin) and probed with the appropriate primary and secondary antibodies. The membrane antigen-antibody complexes were identified using improved chemiluminescent detection (Thermo Fisher Scientific Supersignal West Pico Plus).

### Gelatin zymography

A gelatin zymography assay was used to measure MMP-2 activity. Equal amounts of cell supernatant medium and nonreducing sample buffer were combined, and the mixture was electrophoresed on a 10% SD-PAGE gel that contained 0.1% gelatin. After electrophoresis, the gels were washed with 2.5% Triton X-100 solution for at least one hour at room temperature. Afterward, the gels were incubated for an additional night in a reaction solution containing 0.05% Brij-35, 10 mM CaCl2, 200 mM NaCl, and 50 mM Tris-HCl (pH 7.5). After the gels were stained with 0.05% Coomassie Brilliant Blue solution, they were destained with 40% methanol and 10% acetic acid. Unstained bands represented the gelatinolytic activity (MMP-2 activity), which was quantified using NIH ImageJ software.

### Intraperitoneal Injections

IL-33^−/−^ mice were subjected to intraperitoneal (i.p.) injections with 4 μg of IL-33_95–270_, IL-33_99–270_, IL-33_109–270_ or PBS at P12, P13, and P14.

### Oxygen-induced retinopathy (OIR)

OIR was induced as reported by Smith et al.^27^ and quantified using the method of Connor et al.^28^. At P7, the pups and dams were exposed to hyperoxia (75% oxygen) for 5 days (P7 to P12), and on P12, the pups and dams were returned to room air. Pups of the same age that were housed in room air were used for the control group. At P17, the pups were sacrificed, the eyes were enucleated, and the retinas were isolated and stained with the endothelial cell marker isolectin B4 for 24 h at room temperature. Then, the retinas were washed, flat-mounted and observed under a confocal microscope (Zeiss LSM 800). The captured images were then assessed for retinal neovascularization (%) utilizing Nikon NIS-Elements Advanced Research Software (neovascularization (%) = fluorescence intensity from the highlighted region/total fluorescence intensity × 100). NIH ImageJ software was used to calculate the avascular area percentage (avascular area/total retinal area × 100).

### Immunofluorescence staining

Immunofluorescence staining was performed on retinal cross sections as described previously^25^. The mouse pups were subjected to OIR, and at P15, they were euthanized, and their eyes were enucleated and embedded in optimal cutting temperature (OCT) compound. The cryosections made from the central retina were fixed, permeabilized, blocked, and stained with rat anti-mouse CD31 (1:100) and rabbit anti-mouse Ki-67 (1:200) primary antibodies overnight at 4 °C. The sections were washed and stained with Alexa Fluor 568-conjugated anti-rabbit and 488-conjugated anti-rat secondary antibodies. These sections were examined and imaged using a confocal microscope (Zeiss LSM800), and the cells that stained for both Ki-67 and CD-31 were counted as proliferating endothelial cells.

### RNA sequencing

Quiescent HRMVECs were treated with IL-33_95–270_, IL-33_99–270_, IL-33_109–270_ and IL-33_112–270_ for 24 h. TRIzol reagent was used to extract total cellular RNA, which was then sent for mRNA sequencing. Gene expression was determined by creating 3′ mRNA-seq libraries with Lexogen’s Quant-seq Kit and sequencing (1 × 75 bp) on a NovaSeq 6000. STAR was used to align sequencing data to the mouse genome (mm10) before tabulating counts across genes (htseq-count). EdgeR was used to identify differentially expressed genes, and heatmaps were created using the R program’s WGCNA package^29^ and modifying the R-codes according to a method outlined by Johnstone^30^. The mRNA sequencing data were uploaded to the Gene Expression Omnibus (code GSE245784).

### Statistics and reproducibility

Three independent experiments with multiple replicates were carried out, and the results are presented as the mean ± SD. We utilized a two-tailed *t-*test to compare the differences between the two groups. One-way ANOVA with Tukey’s correction was used when comparing more than two groups in an experiment. GraphPad Prism 9 was utilized for statistical analysis. All *p* values < 0.05 indicated statistical significance.

## Results

### Cloning, expression, and purification of recombinant IL-33 proteins

**Fig. 2:**
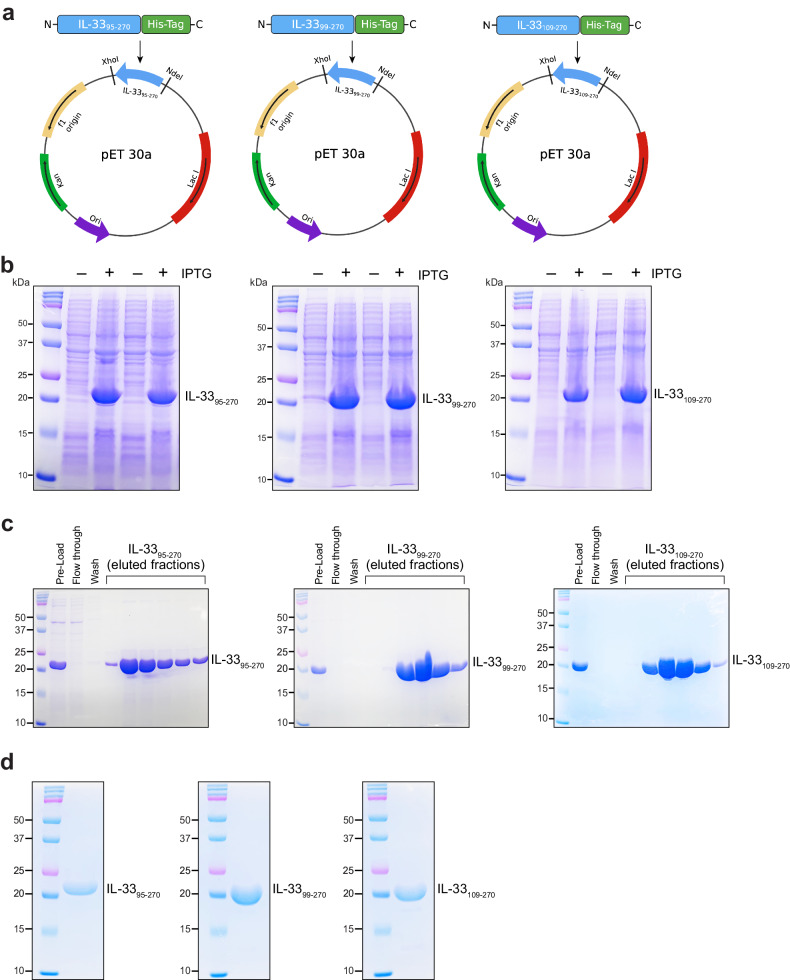
Cloning, expression, and purification of IL-33 constructs. **a** Restriction map of the cloned IL-33 constructs in the pET30a vector. **b** The positive clones for each IL-33 construct were grown, induced with IPTG, and analyzed via 12% SDS‒PAGE. **c** SDS‒PAGE profile of the recombinant IL-33_95–270_, IL-33_99–270_, and IL-33_109–270_ proteins after Ni-NTA column purification. **d** SDS‒PAGE profile of purified recombinant IL-33 proteins after dialysis.

Human mast cell proteases (Chymase and Granzyme B) and neutrophil proteases (Elastase and Cathepsin G) have been shown to produce mature forms of IL-33 (IL-33_95–270_, IL-33_99–270_, IL-33_109–270_ and IL-33_111–270_), which are approximately thirty times more potent than full-length IL-33_1-270_ in eliciting type 2 cytokines, eosinophils, and group 2 innate lymphoid cells (Fig. 1)^19,20^. Therefore, proteolytic processing is crucial for controlling IL-33 activity during inflammation. Neutrophils and mast cell proteases disturb the balance of proangiogenic and antiangiogenic factors, contributing to pathological angiogenesis in proliferative retinopathies. Therefore, to understand the significance of the mature form of IL-33 in angiogenesis, we first cloned, expressed, and purified human IL-33_95–270_, IL-33_99–270_, and IL-33_109–270_ proteins. The various human IL-33 DNA fragments were amplified by polymerase chain reaction (PCR) and ligated to the pET30a plasmid using Xhol and Ndel restriction sites (Fig. 2a). The pET30a vectors containing the DNA constructs were transformed into competent DH5α cells, which were subsequently cultured on kanamycin-containing LB agar plates. Nucleotide sequencing verified the positive clones, which were then transformed into *E. coli* [strain BL-21 (DE3)]. One positive colony for each construct was cultured in 5 ml of LB broth and induced with 1 mM IPTG. SDS‒PAGE was performed to examine IPTG-induced and uninduced *E. coli* cell lysates (Fig. 2b). Each recombinant IL-33 construct carried a 6X histidine tag at the carboxy terminus; therefore, purification of the recombinant IL-33s was carried out utilizing affinity chromatography with Ni-NTA resin. SDS‒PAGE was used to evaluate each eluted fraction (Fig. 2c). The eluted fractions were pooled and examined by SD-PAGE (Fig. 2d).

### A mature form of IL-33 (IL-33_99-270_) generated by neutrophil elastase is able to induce angiogenic events in human retinal endothelial cells

**Fig. 3:**
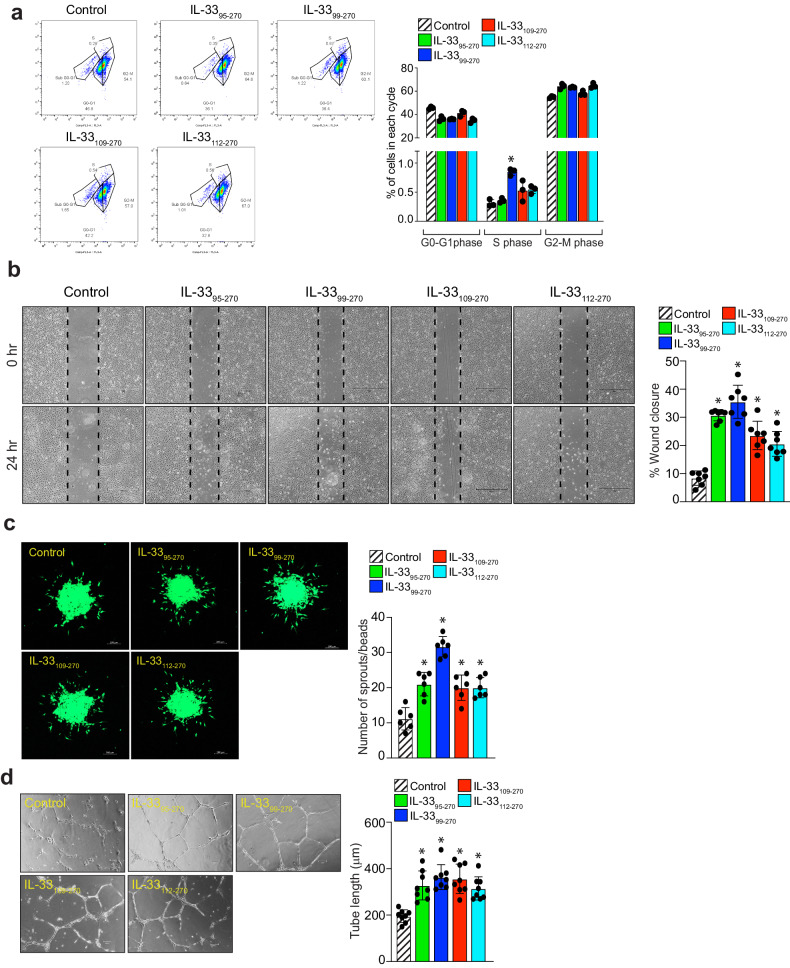
IL-33_99–270_ is a more potent regulator of angiogenic events compared to other IL-33 mature forms. **a** Quiescent HRMVECs were treated with or without IL-33_95–270_, IL-33_99–270_, IL-33_109–270_, and IL-33_112–270_ (20 ng/mL), and cell proliferation was measured by a BrdU cell proliferation assay. **b** Cell migration was measured using a wound healing assay using all the same conditions as described in (**a**). **c** A spheroid assay was used to evaluate sprouting in HRMVECs, and images were captured after 2 days under a Zeiss LSM800 microscope. **d** Tube formation was assessed using growth factor-reduced Matrigel using the all the same conditions as described in (**a**). The bar graphs show the quantitative analysis of 3 independent experiments, expressed as the mean ± SD. *p < 0.05 vs. control. The scale bar represents 200 μm in (**c**).

To understand the functional significance of recombinant IL-33s in angiogenic events, we next studied the effects of IL-33_95–270_, IL-33_99–270_, IL-33_109–270_ and IL-33_112–270_ on HRMVEC proliferation, migration, sprouting, and tube formation. IL-33_112–270_ was purchased from R&D Systems (Minneapolis, MN). A FITC-BrdU flow cytometry assay was used to evaluate the effect of recombinant IL-33s on proliferation. We observed a significant increase in HRMVEC proliferation induced by IL-33_99–270_, with little or no increase in proliferation induced by the other recombinant IL-33s (Fig. 3a). The effect of recombinant IL-33s on the migration of HRMVECs was observed using a wound healing assay. IL-33_95–270_, IL-33_99–270_, IL-33_109–270_, and IL-33_112–270_ increased the migration of HRMVECs (Fig. 3b). The effect of the recombinant IL-33s on tip cell formation/sprouting was assessed using a spheroid assay. IL-33_95–270_, IL-33_99–270_, IL-33_109–270_, and IL-33_112–270_ increased tip cell formation/sprouting in HRMVECs (Fig. 3c). A 2-dimensional Matrigel assay was used to assess tube formation in HRMVECs, which showed that IL-33_95–270_, IL-33_99–270_, IL-33_109–270_, and IL-33_112–270_ were able to induce tube formation (Fig. 3d). These observations suggested that the IL-33_99–270_ generated by neutrophil elastase was able to induce all angiogenic events in HRMVECs.

### IL-33_99-270_ is more potent than other recombinant IL-33s at activating angiogenic pathways in human retinal endothelial cells

**Fig. 4:**
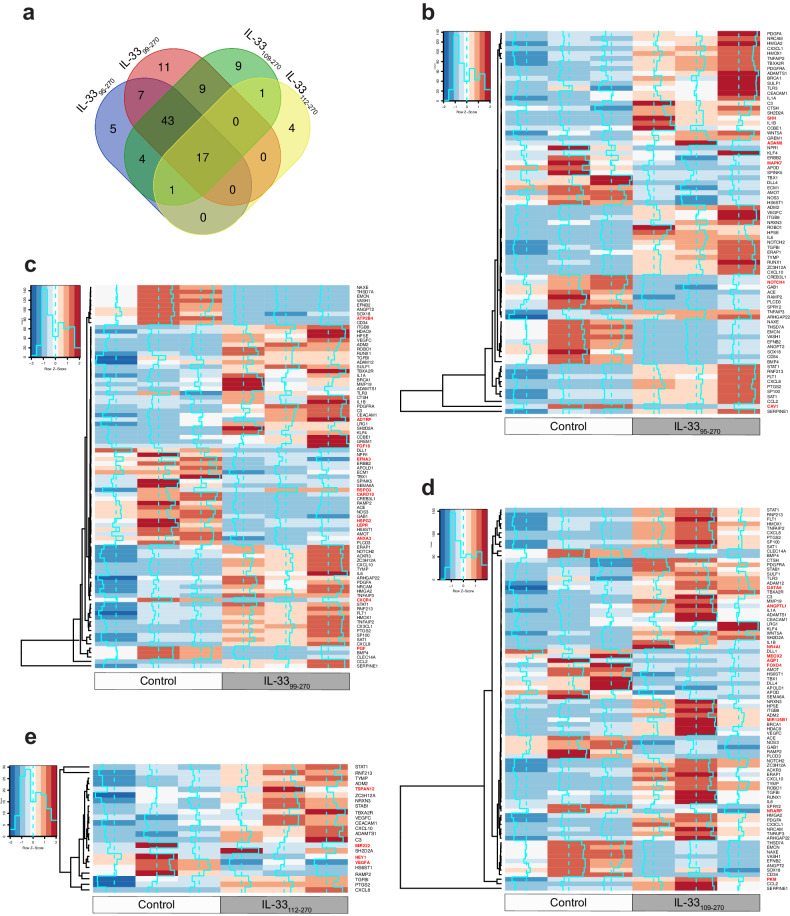
The mature forms of IL-33 differentially regulate angiogenic genes. Quiescent HRMVECs were treated with or without IL-33_95–270_, IL-33_99–270_, IL-33_109–270_, and IL-33_112–270_ (20 ng/mL), and total cellular RNA was extracted and subjected to mRNA sequencing. **a** Venn diagram showing the number of angiogenic genes regulated by IL-33_95–270_, IL-33_99–270_, IL-33_109–270_, and IL-33_112–270_ compared to the control. **b** Heatmap showing the expression profile of angiogenic genes regulated by IL-33_95–270_ in HRMVECs. **c** Heatmap showing the expression profile of angiogenic genes regulated by IL-33_99–270_ in HRMVECs. **d** Heatmap showing the expression profile of angiogenic genes regulated by IL-33_109–270_ in HRMVECs. **e** Heatmap showing the expression profile of angiogenic genes regulated by IL-33_112–270_ in HRMVECs. The differentially expressed genes in the heatmap that were regulated only by specific mature versions of IL-33 are highlighted in red.

To understand how these recombinant IL-33s influence angiogenic processes in HRMVECs, we treated quiescent HRMVECs with the recombinant IL-33s for 24 h, extracted the RNA, and performed 3′ RNA sequencing. Comparative analysis of the regulated angiogenic genes distinctly revealed that the majority of the angiogenic genes were regulated by IL-33_99–270_ (Fig. 4a). Furthermore, heatmaps generated using the angiogenic genes differentially expressed between the recombinant IL-33 groups and the control group showed that IL-33_95–270_ regulated 77 angiogenic genes (Fig. 4b), IL-33_99–270_ regulated 87 angiogenic genes (Fig. 4c), IL-33_109–270_ regulated 84 angiogenic genes (Fig. 4d) and IL-33_112–270_ regulated 23 angiogenic genes (Fig. 4e) in HRMVECs. The differentially expressed genes in the heatmaps that were distinct for each IL-33 mature form are highlighted in red.

**Fig. 5:**
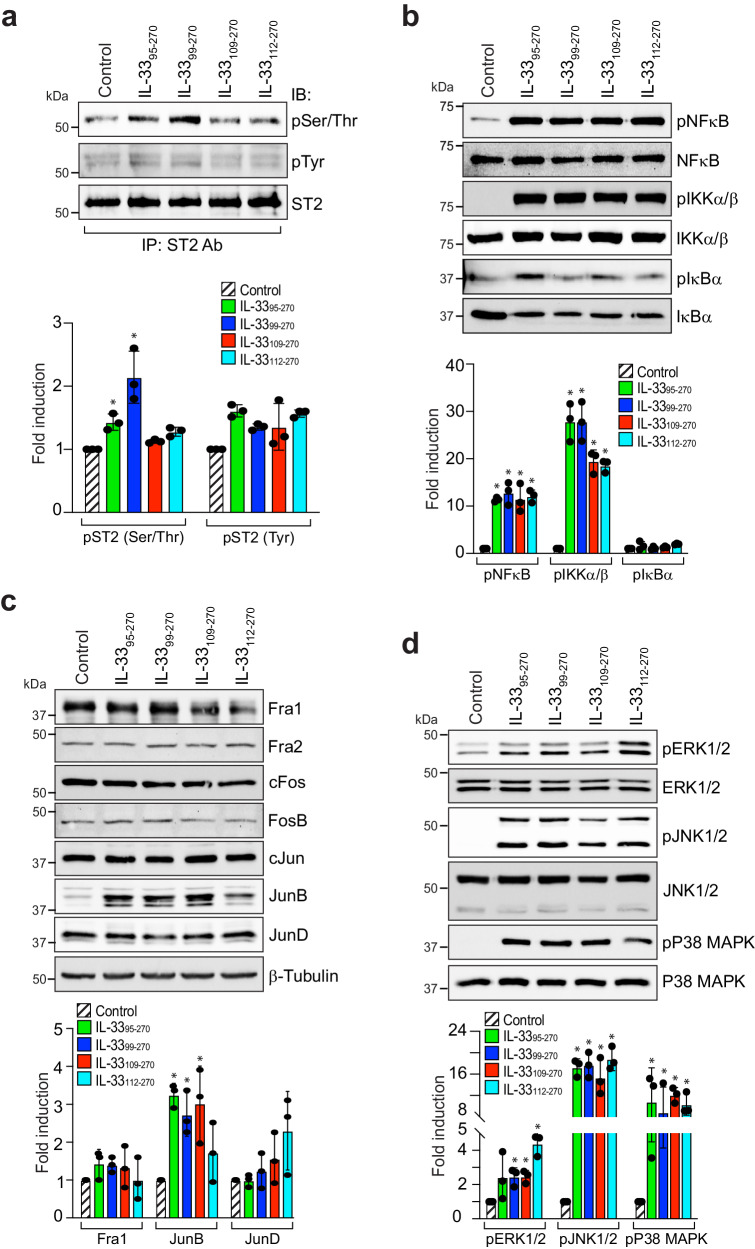
Differential regulation of transcription factors and MAP kinase by mature forms of IL-33. **a** Quiescent HRMVEC monolayers were treated with IL-33_95–270_, IL-33_99–270_, IL-33_109–270_, or IL-33_112–270_ for 5 min, and cell extracts were prepared. Equal amounts of cell extracts were immunoprecipitated (IP) with an anti-ST2 antibody, and the immunocomplexes were analyzed by Western blotting (WB) with anti-phosphotyrosine (pTyr) and anti-phospho serine/threonine (pSer/Thr) antibodies. The blot was reprobed with anti-ST2 antibodies for lane loading control. **b**, **d** Quiescent HRMVEC monolayers were treated with IL-33_95–270_, IL-33_99–270_, IL-33_109–270_, or IL-33_112–270_ for 30 min, and cell extracts were prepared and analyzed by Western blotting for the indicated proteins. **c** Quiescent HRMVEC monolayers were treated with IL-33_95–270_, IL-33_99–270_, IL-33_109–270_, or IL-33_112–270_ for 4 h, and cell extracts were prepared and analyzed by Western blotting for the indicated proteins, followed by normalization to β-tubulin. The bar graphs show the quantitative analysis of 3 independent experiments, expressed as the mean ± SD. *p < 0.05 vs. control.

IL-33 is a functional ligand of its receptor, ST2. Therefore, we next studied the effect of these recombinant IL-33s on ST2 activation in HRMVECs. All the recombinant IL-33s induced the serine/threonine (Ser/Thr) phosphorylation of ST2, but a significant effect was observed only in IL-33_99–270_-treated cells (Fig. 5a). These recombinant IL-33s had little or no effect on the tyrosine (Tyr) phosphorylation of ST2 in HRMVECs (Fig. 5a). Our previous studies revealed that IL-33 regulates NF-κB activation and JunB expression in retinal endothelial cells^7^. Therefore, we investigated the effect of the recombinant IL-33s on NF-κB signaling and proto-oncogene expression in HRMVECs. We observed that all recombinant IL-33s induced the phosphorylation of NF-κB and IKKα/β, with little or no effect on Iκbα phosphorylation (Fig. 5b). We also observed that the recombinant IL-33s induced JunB phosphorylation in HRMVECs but had little or no effect on other proto-oncogenes (Fig. 5c). Mitogen-activated protein kinases (MAPKs), particularly extracellular signal-regulated kinases 1/2 (ERK1/2), Jun amino (N)-terminal kinases 1/2 (JNK1/2), and p38 MAPK, have been shown to regulate various physiological processes, including angiogenesis^31^. We observed that the recombinant IL-33s induced the phosphorylation or activation of ERK1/2, JNK1/2, and p38 MAPK in HRMVECs (Fig. 5d).

**Fig. 6:**
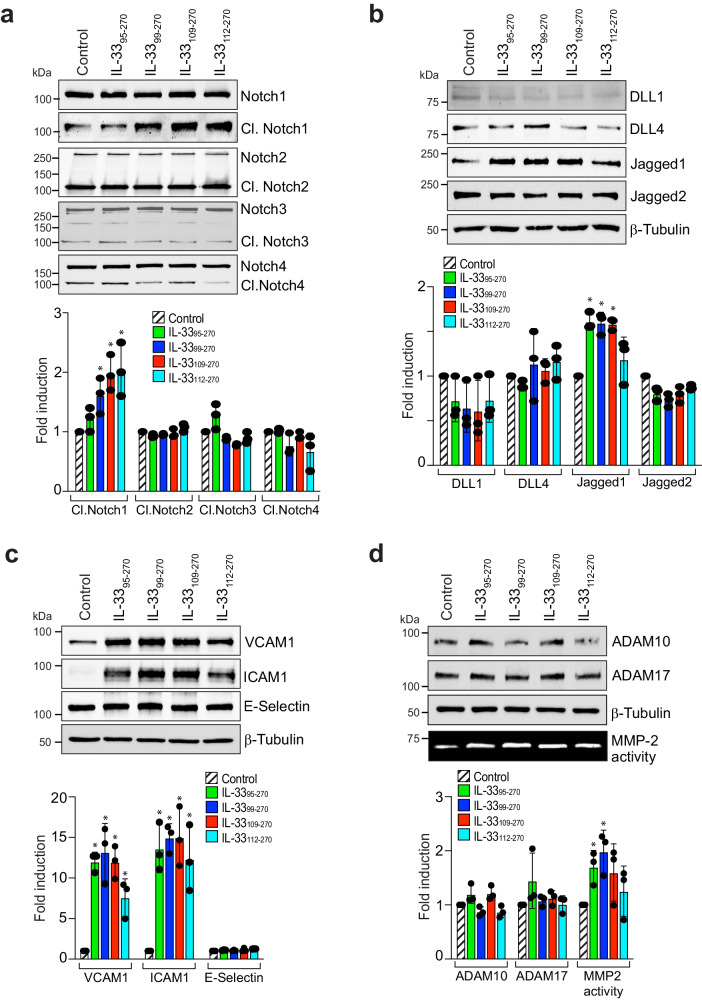
IL-33_95–270_ and IL-33_99–270_ are potent regulators of EC activation and MMP-2 activity. **a**–**c** Quiescent HRMVEC monolayers were treated with IL-33_95–270_, IL-33_99–270_, IL-33_109–270_, or IL-33_112–270_ for 4 h, and cell extracts were prepared and analyzed by Western blotting for the indicated proteins, followed by normalization to β-tubulin. **d** Quiescent HRMVEC monolayers were treated with IL-33_95–270_, IL-33_99–270_, IL-33_109–270_, or IL-33_112–270_ for 24 h, and the supernatant media and cell lysates were prepared. The cell lysates were analyzed by Western blotting for active ADAM10 and ADAM17 levels using specific antibodies, followed by normalization to β-tubulin. Gelatin zymography was performed to analyze MMP-2 activity in the supernatant media. The bar graphs show the quantitative analysis of 3 independent experiments, expressed as the mean ± SD. *p < 0.05 vs. control.

The initiation of the angiogenic cascade requires the activation of specific signaling pathways that promote endothelial cell invasion of the underlying stroma and the formation of vascular sprouts. Notch ligands and receptors regulate angiogenesis^32^. Therefore, we investigated the effect of these recombinant IL-33s on the expression of Notch receptors and ligands in HRMVECs. We observed that all these recombinant IL-33s increased the levels of cleaved Notch1 and Jagged1 in HRMVECs (Fig. 6a, b). We also investigated the effect of the recombinant IL-33s on signaling molecules responsible for endothelial cell activation and angiogenesis. All recombinant IL-33s significantly induced the expression of ICAM1 and VCAM1 in HRMVECs, with little or no effect on E-selectin (Fig. 6c). Extracellular proteases such as matrix metalloproteases (MMPs) and ADAMs facilitate sprout formation and invasion of connective tissue through controlled degradation of extracellular matrix proteins (ECMs)^33,34^. Mutations in ADAMTS10 and ADAMTS17 reportedly result in Weill–Marchesani syndrome (an eye anomaly)^35^. Furthermore, increased plasma MMP-2 levels were also reported in patients with proliferative diabetic retinopathy^36^. Therefore, we investigated the effect of the recombinant IL-33s on ADAM10, ADAM17, and MMP-2 activation in HRMVECs (Fig. 6d). To our surprise, we observed that MMP-2 activity was induced by the recombinant IL-33s in HRMVECs and that the greatest increase in MMP-2 activity was observed in IL-33_99–270_-treated cells (Fig. 6d).

### IL-33_99-270_ is a potent regulator of retinal neovascularization in a murine model of oxygen-induced retinopathy

**Fig. 7:**
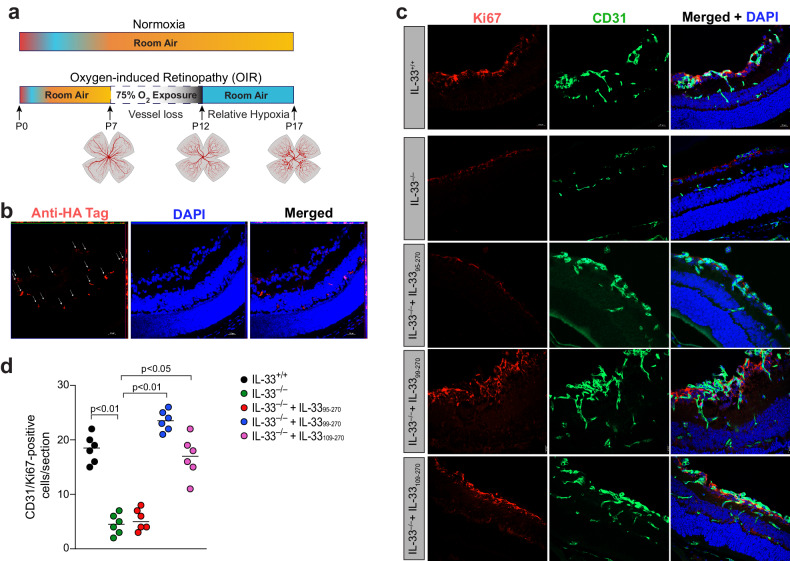
IL-33_99–270_ and IL-33_109–270_ regulate OIR-induced retinal EC proliferation. **a** Schematic diagram showing the murine OIR model. C57BL/6 mouse pups with dams were exposed to 75% oxygen from P7 to P12 and returned to the room at P12. The P12–P17 phase is the neovascular phase. **b** C57BL/6 mouse pups with dams were exposed to 75% oxygen from P7 to P12 and returned to the room at P12. The mouse pups were subjected to intraperitoneal (i.p.) injection with PBS, recombinant IL-33_95–270_, IL-33_99–270_ or IL-33_109–270_ at P12, P13, and P14. At P15, the eyes were enucleated, the retinas were isolated, and cryosections were prepared and stained with anti-HA tag antibodies. **c** IL-33^+/+^ and IL-33^−/−^ pups were subjected to i.p. injection with PBS or the mature forms of IL-33, and at P15, the retinas were isolated and fixed, and cross-sections were made and subjected to immunofluorescence staining for CD31 and Ki67. **d** Retinal EC proliferation was measured by counting CD31- and Ki67-positive cells that extended anteriorly to the inner limiting membrane per section (*n* = 6 eyes, 3 sections/eye). The scale bar represents 20 μm in (**b**, **c**).

Based on our findings in Figs. 4–6, we concluded that IL-33_95–270_, IL-33_99–270_, and IL-33_109–270_ not only significantly increased the expression of various angiogenic genes but also induced angiogenic signaling pathways in retinal endothelial cells, whereas IL-33_112–270_ had only a modest effect. Therefore, we specifically studied the effects of IL-33_95–270_, IL-33_99–270_, and IL-33_109–270_ on retinal neovascularization in a mouse model of oxygen-induced retinopathy (OIR) (Fig. 7a). The neovascular phase at P12-P17 in this model reflects the second phase of retinopathy of prematurity (ROP) and certain symptoms of proliferative diabetic retinopathy. Wild-type (WT) mice at P12, P13, and P14 were subjected to intraperitoneal (i.p.) injections with the recombinant IL-33s, and the retinas were analyzed at P15 for the presence of the recombinant IL-33s. Since these recombinant IL-33s were HA-tagged, retinal sections at P15 were stained with anti-HA tag antibodies to detect the presence of these recombinant proteins. We observed florescence in all layers of the retina, suggesting the presence of these recombinant IL-33s in the retina after i.p. injection (Fig. 7b). WT (IL-33^+/+^) and IL-33^–/–^ mice were injected i.p. with IL-33_95–270_, IL-33_99–270_, IL-33_109–270_ or PBS at P12, P13, and P14. At P15, the eyes were enucleated, and cryosections were prepared and stained for CD31 and Ki67 to observe retinal endothelial cell (EC) proliferation (Fig. 7c). IL-33 depletion reduced OIR-induced retinal EC proliferation, and i.p. injection of IL-33_99–270_ or IL-33_109–270_ significantly restored OIR-induced EC proliferation in the ischemic retina of IL-33^–/–^ mice (Fig. 7d). The i.p. injection of IL-33_95–270_ failed to rescue retinal EC proliferation in IL-33^–/–^ mice (Fig. 7d).

**Fig. 8:**
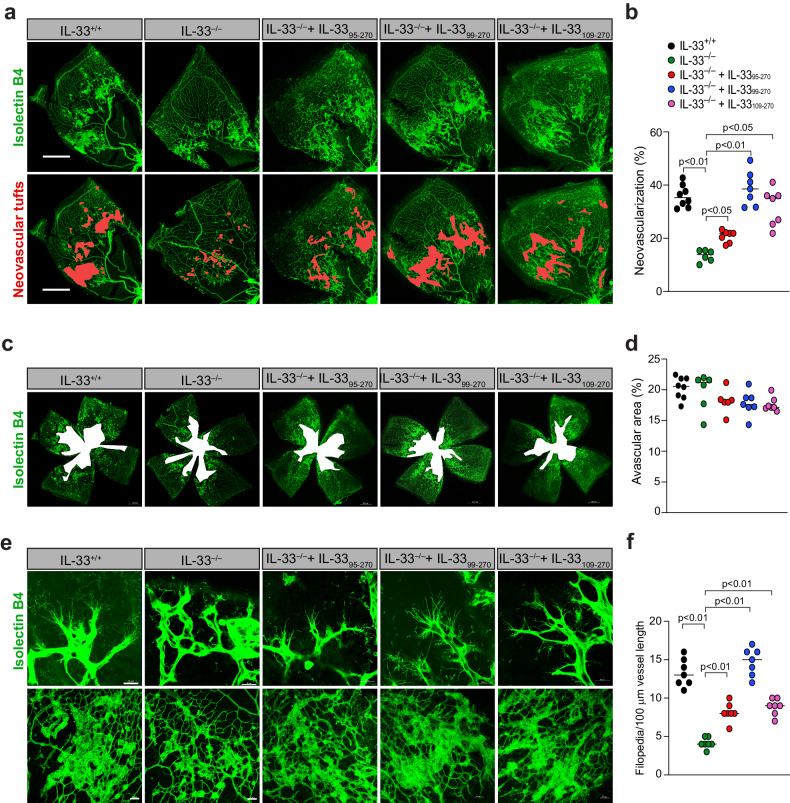
IL-33_99–270_ and IL-33_109–270_ regulate OIR-induced retinal neovascularization. **a**, **c**, **e** IL-33^+/+^ and IL-33^−/−^ pups were subjected to i.p. injection with PBS, recombinant IL-33_95–270_, IL-33_99–270_ or IL-33_109–270_ at P12, P13, and P14. At P17, the eyes were enucleated, and the retinas were isolated, fixed, and stained with isolectin B4. Flat mounts were prepared and examined for retinal neovascularization (**a**), avascular areas (**c**), and endothelial tip cell formation (**e**). Neovascularization is highlighted in red in (**a**). The bar graphs represent the quantitative analysis of neovascularization (**b**), avascular areas (**d**), and number of sprouts/100 mm vessel length (**f**). There were *n* = 8 (**b**, **d**, **f**) biologically independent eyes per group, and the data are expressed as the mean ± SD. The scale bars represent 500 μm in (**a**, **c**), 20 μm in (**e**) in the upper row, and 50 μm in (**e**) in the lower row.

We also investigated the role of these recombinant IL-33s in retinal EC filopodium formation, neovascularization, and vaso-obliteration. WT (IL-33^+/+^) and IL-33^–/–^ mice were subjected to i.p. injection with IL-33_95–270_, IL-33_99–270_, IL-33_109–270_ or PBS at P12, P13, and P14. No discernible phenotypic changes were observed in the animals following the injection of the recombinant IL-33s. At P17, the retinas were isolated, fixed and stained with isolectin B4. We observed a significant decrease in retinal EC filopodium formation and neovascularization in IL-33^–/–^ mice compared to IL-33^+/+^ mice (Fig. 8a, b, e, f). The intraperitoneal injection of IL-33_95–270_, IL-33_99–270_, and IL-33_109–270_ significantly restored retinal EC filopodium formation and neovascularization in the ischemic retina of IL-33^–/–^ mice (Fig. 8a, b, e, f). IL-33 depletion had no significant effect on OIR-induced vaso-obliteration, as we were unable to observe any effect of IL-33 knockdown or the recombinant IL-33s on the avascular area in the ischemic retina (Fig. 8c, d).

## Discussion

In the present study, we showed that HRMVEC proliferation, migration, sprouting, and tube formation were impacted differently by the mature versions of IL-33s produced by neutrophil and mast cell serine proteases. Additionally, these mature versions of IL-33 exhibited great potency in triggering the activation of endothelial cells and inducing signaling pathways involved in EC angiogenesis. We further showed that the mature form of IL-33 generated by the neutrophil serine protease elastase was the most effective at activating angiogenic genes in human retinal endothelial cells. We showed that genetic deletion of IL-33 reduced OIR-induced retinal neovascularization and that intraperitoneal injections of mature versions of IL-33_99–270_ and IL-33_109–270_ produced by neutrophil and mast cell serine proteases restored retinal neovascularization in IL-33-depleted ischemic retinas. IL-33_99–270_ was more effective than IL-33_109–270_ in rescuing OIR-induced retinal EC proliferation, sprouting and neovascularization.

Various studies have shown that IL-33 acts as an alarmin or endogenous danger signal, alerting the immune system to cell or tissue damage^37^. IL-33 is constitutively expressed in the nucleus of epithelial and endothelial cells in human and mouse tissues and contains a helix-turn-helix domain, which allows it to bind to DNA; upon cellular injury, it is released into the extracellular space^37^. The exact function of IL-33 in the nucleus is poorly understood, yet amino acids 40-58 in human IL-33 are responsible for histone binding and nuclear localization^13–15^. The nuclear localization domain suppresses the cytokine activity of IL-33, as evidenced by the greater biological activity of the mature versions of the protein compared to the full-length version^20^. It was first believed that full-length IL-33 was an inactive precursor of the cytokine that needed to be cleaved and activated by caspase 1/inflammasomes before it could be biologically active^3^. However, various studies have demonstrated that IL-33 cleavage by caspase-1 inactivates IL-33^2,38^. In contrast to full-length IL-33, caspase 3- and 7-mediated cleavage products of human IL-33 do not produce significant ST2-dependent responses^16^. Thus, caspase-mediated cleavage of the IL-33 protein results in its deactivation during apoptosis.

Cleavage of IL-33 by caspases results in its inactivation, but it has also been demonstrated that cleavage of IL-33 in its central domain by a variety of inflammatory cell-derived proteases activates IL-33^19^. Recently, it was discovered that full-length IL-33 functions as a protease sensor ready to be cleaved in its “activation” domain by various proteases^39^. The cleavage products of 18–21 kDa (mature forms) are produced when neutrophil elastase and cathepsin G, as well as mast cell chymase and tryptase, cleave full-length IL-33^19,20^. Compared to full-length IL-33, these mature forms exhibit approximately 30- to 60-fold greater biological activity^19,20,40^. Cleavage within the central domain of IL-33 may have an important function, as it liberates the C-terminal IL-1-like cytokine domain from the inhibitory activity of the N-terminal nuclear localization domain.

It has been demonstrated that the cleavage of IL-33 within its activation domain by inflammatory proteases is required for the type 2 innate immune responses mediated by IL-33^20^. Neutrophil serine proteases play a significant role in regulating the bioactivity of IL-33 in sterile neutrophilic inflammation, which plays a vital role in the pathogenesis of acute ischemia-induced injuries, acute lung and liver injuries, and chronic lung, bowel, and joint diseases^41^. It has been demonstrated that IL-33 orchestrates neutrophil migration into joints^42^ and plays significant roles in animal models of rheumatoid arthritis^43,44^. We and others have demonstrated that IL-33 controls EC activation and angiogenesis in various in vitro and in vivo models^5–7^. However, no attempt has been made to pinpoint the specific functional domain of IL-33 that is primarily responsible for this impact. Because the biological activity of these mature IL-33 variants is 30- to 60-fold greater than that of full-length IL-33, we used human microvascular endothelial cells (HRMVECs) and mouse oxygen-induced retinopathy models to investigate the influence of each of these IL-33 variants on angiogenesis. In the present study, we found that retinal EC migration, sprouting, and tube formation were regulated by all the mature forms of IL-33 produced by neutrophil and mast cell proteases; however, retinal EC proliferation was regulated only by IL-33_99–270_. Our 3′ prime mRNA sequencing results further highlighted the strong potency of neutrophil elastase-generated IL-33_99–270_ in increasing angiogenic gene expression in retinal ECs. Proteolytic cleavage of the pro-IL-33 precursor yields the mature form of IL-33, which binds to its receptor, ST2, to initiate signaling cascades. Our study revealed that IL-33_99–270_ significantly increased ST2 phosphorylation at serine/threonine residues but had little to no effect on tyrosine residues. We found that IL-33_95–270_, IL-33_109–270_, and IL-33_112–270_ did not affect ST2 phosphorylation significantly at serine/threonine or tyrosine residues. The role of IL-33 in ST2 phosphorylation has yet to be determined. However, it is known that IL-33 binds to the ST2 receptor, leading to the recruitment of myeloid differentiation primary response 88 (MyD88) to its intracellular domain and activating NF-κB, MAP kinases, or activator protein 1 (AP-1) pathways^45–48^. Thus, MyD88 might be the link through which these recombinant IL-33s regulate retinal EC signaling. We also observed that all the mature forms of IL-33 significantly induced the activation of NF-κB signaling, MAP kinase signaling, and the expression of AP1 family members.

IL-33 influences the production and function of adhesion molecules, which are essential for cell‒cell adhesion and leukocyte recruitment during inflammation and immunological responses^49^. Therefore, we also investigated the effect of the mature forms of IL-33 on adhesion molecule expression and observed that all the mature forms of IL-33 significantly induced the expression of ICAM1 and VCAM1 in HRMVECs, with little or no effect on E-selectin levels. Furthermore, we also observed that the mature forms of IL-33 induced the activity of MMP-2 (matrix metalloproteinase-2), with a maximum effect shown by IL-33_99–270_. Matrix metalloproteinases (MMPs) are responsible for degrading and remodeling extracellular matrix (ECM) components, regulating developmental and pathological angiogenesis. Therefore, we next investigated the impact of the mature forms of IL-33 on pathological angiogenesis using a murine model of oxygen-induced retinopathy (OIR). We observed that genetic deletion of IL-33 reduced OIR-induced retinal EC proliferation, sprouting and neovascularization and that intraperitoneal injection of IL-33_99–270_ and IL-33_109–270_ significantly rescued OIR-induced proliferation, sprouting and neovascularization in IL-33^–/–^ mice.

In summary, our study identified the differential effects of mature forms of IL-33 produced by neutrophil and mast cell proteases on angiogenic events in HRMVECs and in a murine model of OIR. Our findings revealed two functional domains of IL-33 (amino acids 99–270 and 109–270), which are cleavage products of a mast cell protease (chymase) and neutrophil proteases (elastase and cathepsin G), were crucial for retinal EC activation and retinal neovascularization. Furthermore, we discovered that the neutrophil elastase-cleaved IL-33 domain (IL-33_99–270_) was the most effective at inducing retinal neovascularization in a mouse model of proliferative retinopathy. These findings imply that IL-33 cleavage/activation by neutrophil or mast cell proteases is essential for pathological retinal neovascularization in proliferative retinopathies. These findings provide insight into the regulation of IL-33 activity by inflammatory cells during pathological angiogenesis and may help in the development of future therapeutic strategies for proliferative retinopathies.

## Supplementary information

Supplementary Information

Data File 1

## Data Availability

Supplementary Figs. 1–4 show the uncropped Western blot images used in the article. Supplementary Data 1 (Excel file) contains the statistical source data for all the graphs presented in the article. The raw data will be accessible from the corresponding author upon request. The mRNA sequencing data were uploaded to Gene Expression Omnibus (code GSE245784).
