# Supplementary material for: A neutrophil elastase-generated mature form of IL-33 is a potent regulator of endothelial cell activation and proliferative retinopathy
Source: Exp Mol Med. 2024 Aug 1;56(8):1703–16. doi: 10.1038/s12276-024-01279-y (PMC11372157; doi:10.1038/s12276-024-01279-y)

Supplementary Figure legends

Supplementary Fig. 1
Scans of immunoblots presented in figure 5a and 5b.

Supplementary Fig. 2
Scans of immunoblots presented in figure 5c and 5d.

Supplementary Fig. 3
Scans of immunoblots presented in figure 6a and 6b.

Supplementary Fig. 4
Scans of immunoblots presented in figure 6c and 6d.

Supplementary Fig. 1

Blots presented in Fig. 5a

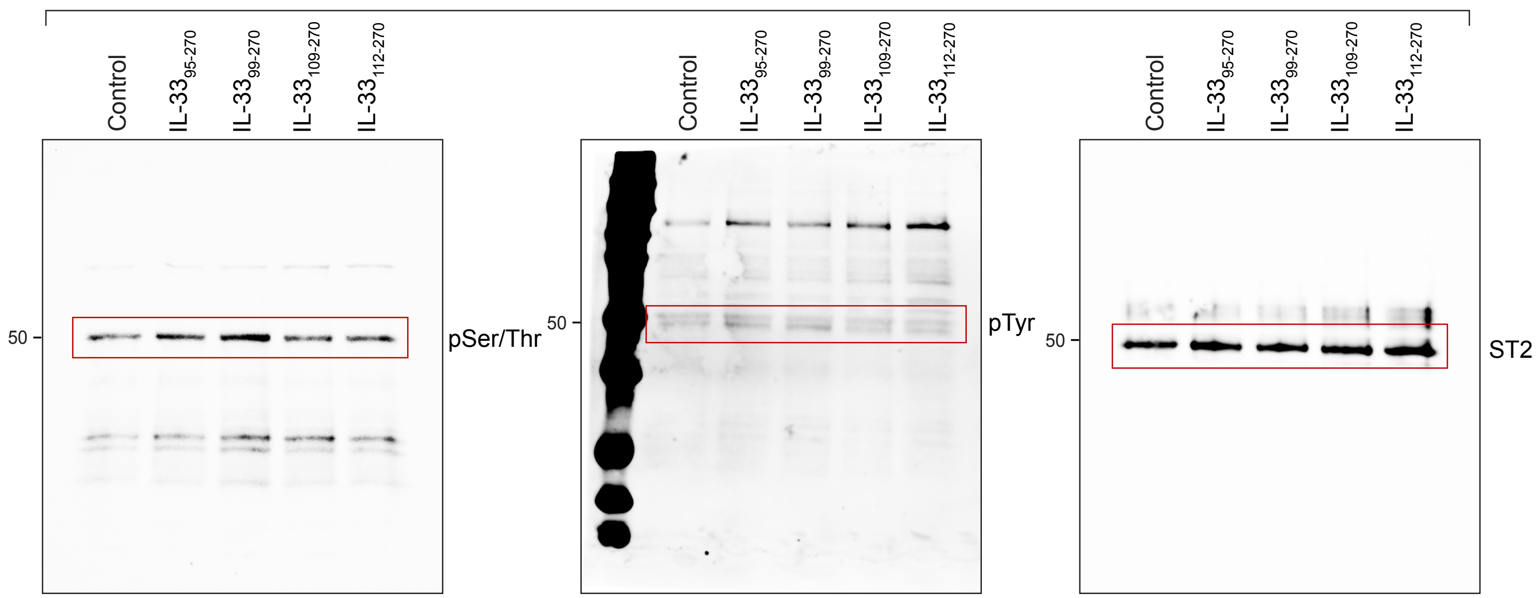

Blots presented in Fig. 5b

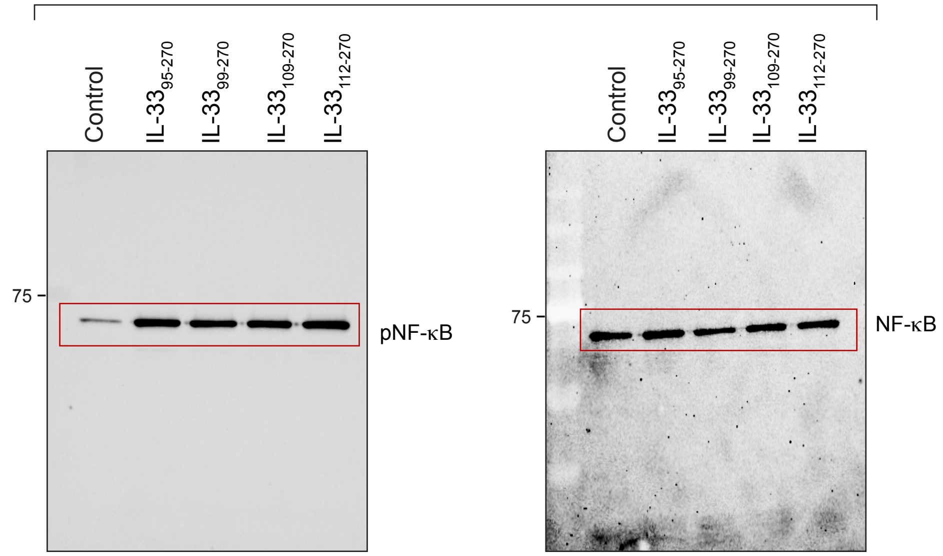

Blots presented in Fig. 5b

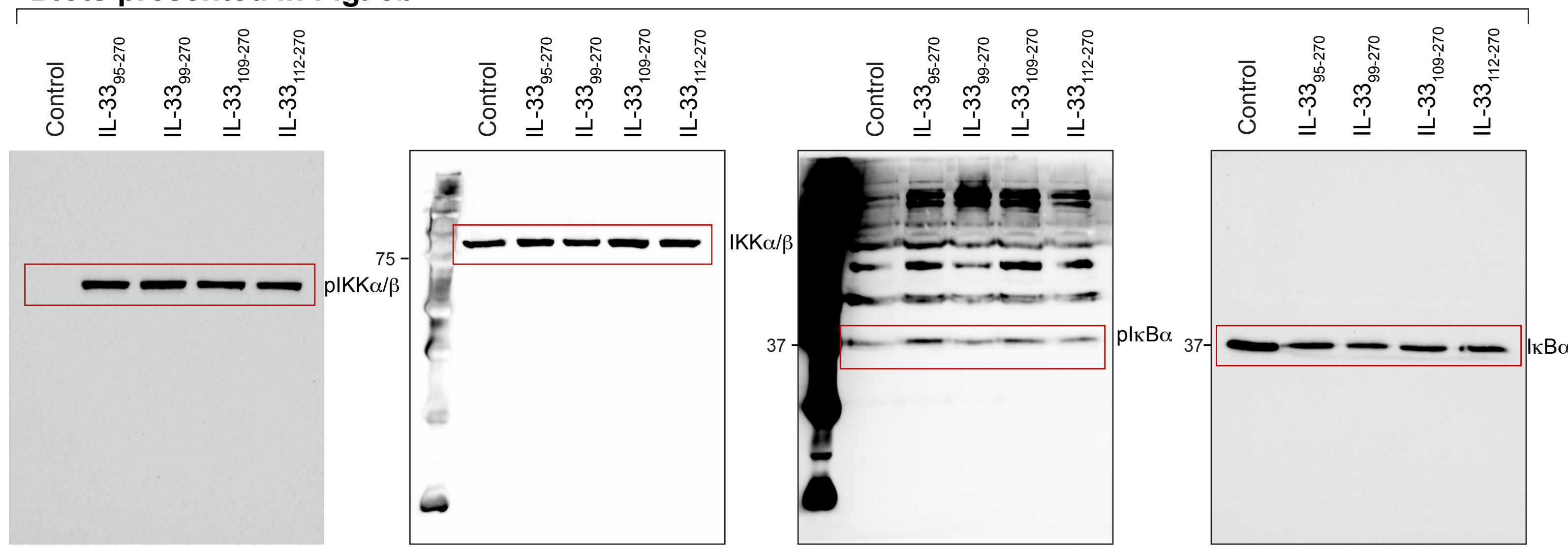

Supplementary Fig. 2

Blots presented in Fig. 5c

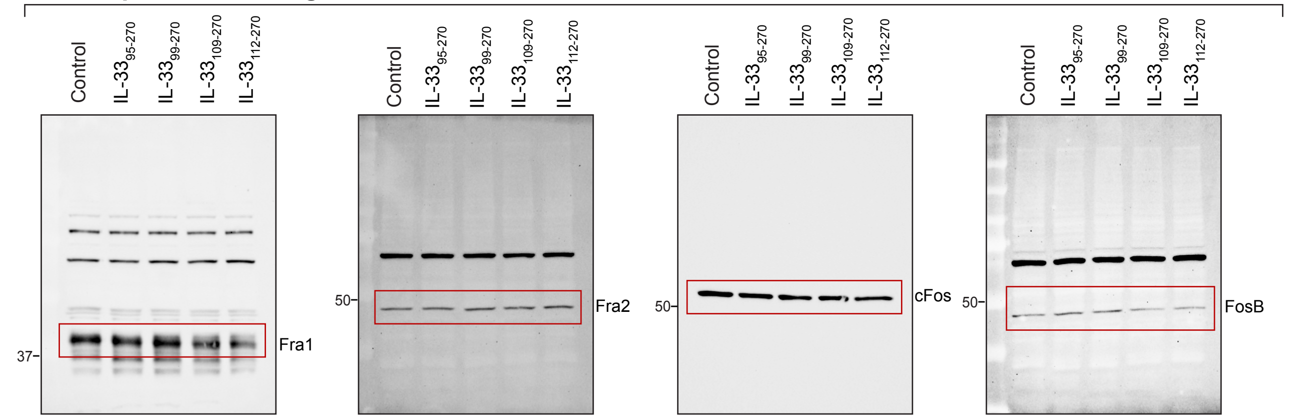

Blots presented in Fig. 5c

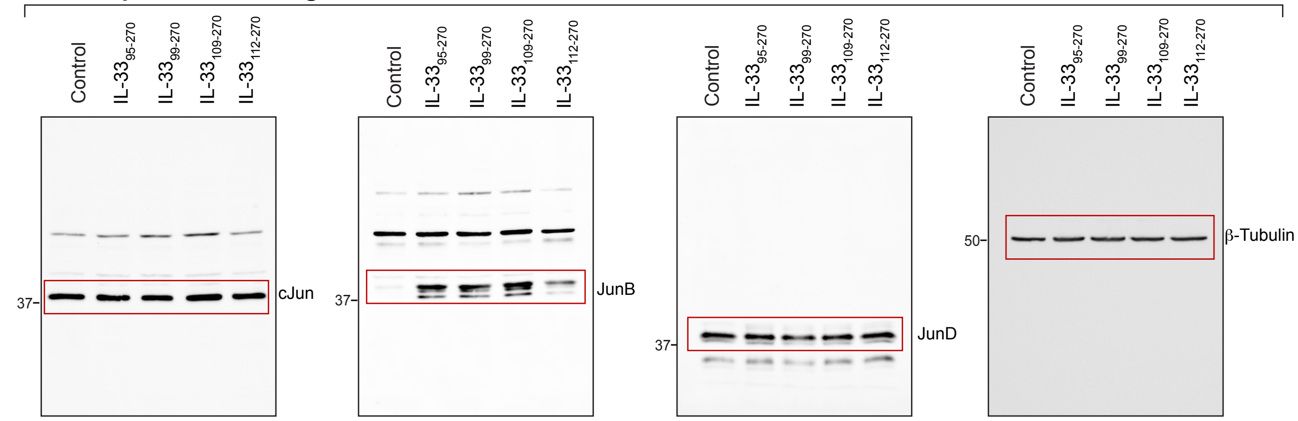

Blots presented in Fig. 5d

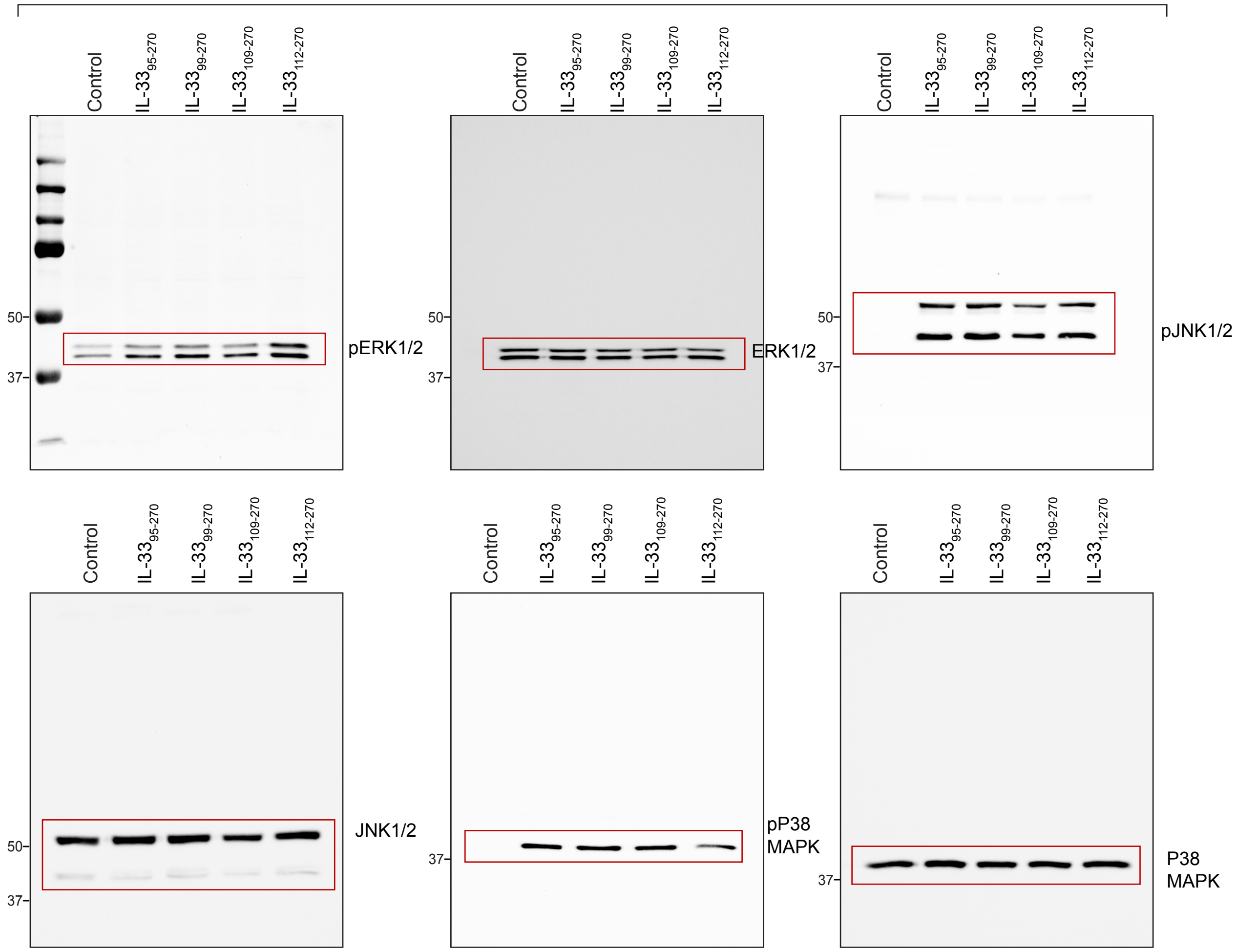

Supplementary Fig. 3

Blots presented in Fig. 6a

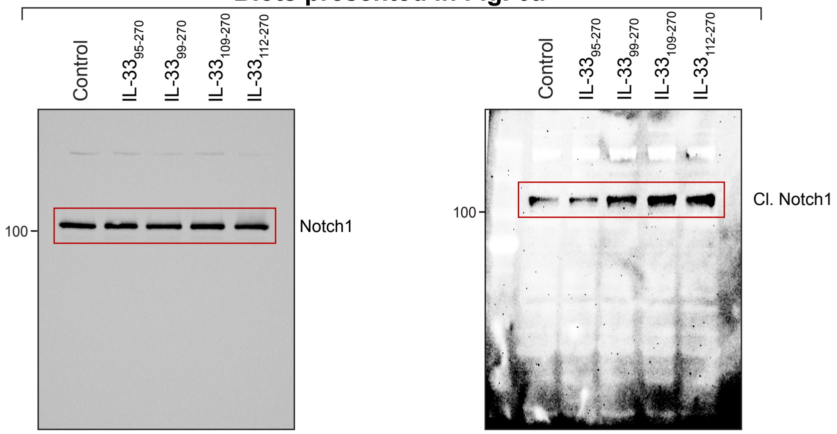

Blots presented in Fig. 6a

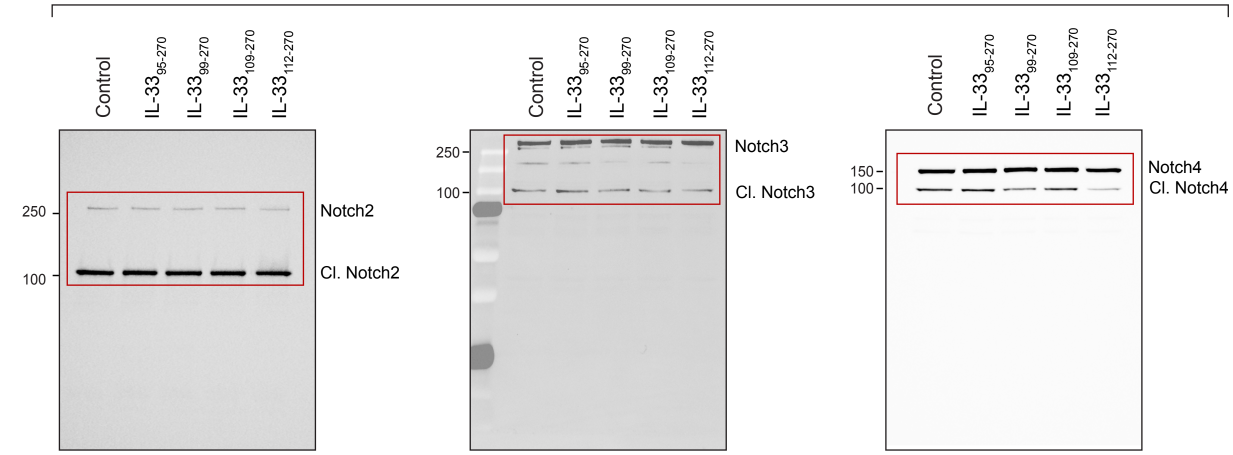

Blots presented in Fig. 6b

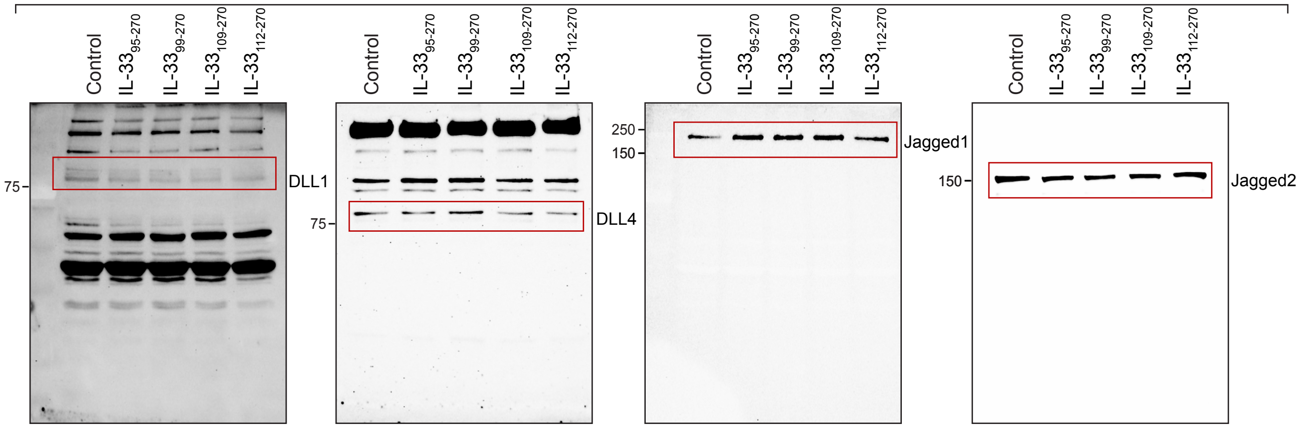

Blots presented in Fig. 6b

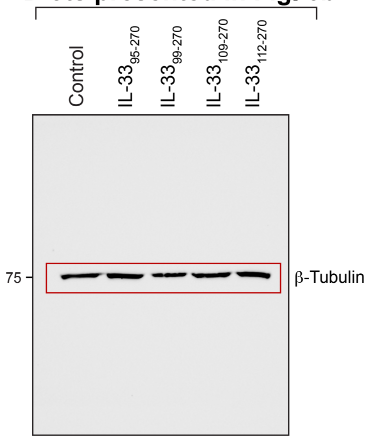

Supplementary Fig. 4

Blots presented in Fig. 6c

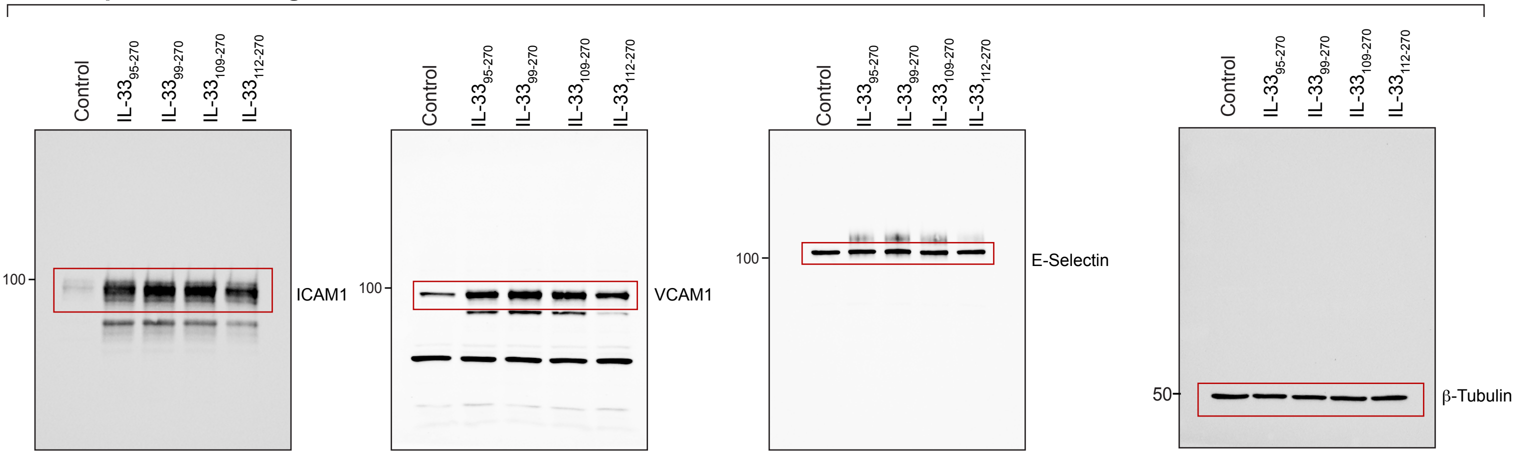

Blots presented in Fig. 6d

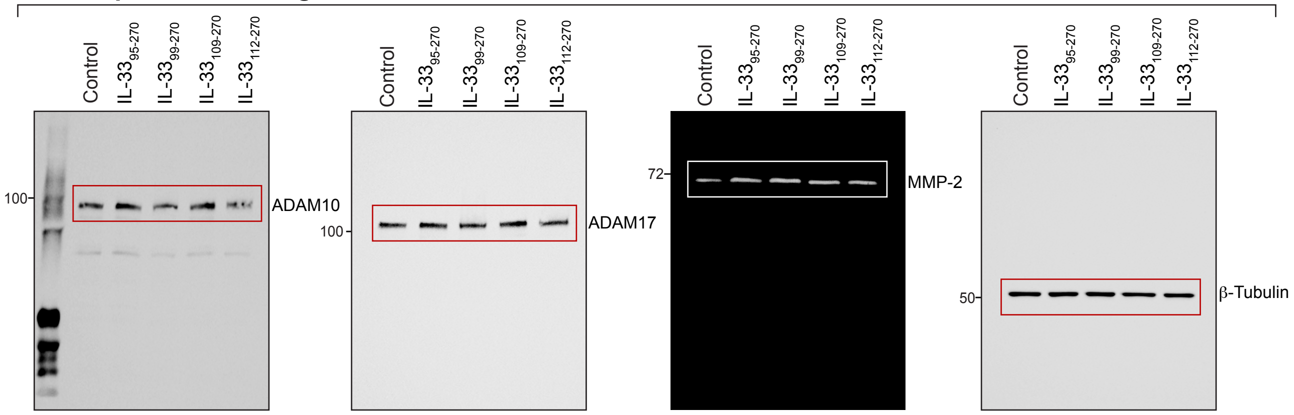

Supplement: Supplementary file 1 — Supplementary Information [file 12276_2024_1279_MOESM1_ESM.pdf]
